# Pharmacogenomics DNA Biomarkers in Colorectal Cancer: Current Update

**DOI:** 10.3389/fphar.2017.00736

**Published:** 2017-10-12

**Authors:** Nurul-Syakima Ab Mutalib, Najwa F. Md Yusof, Shafina-Nadiawati Abdul, Rahman Jamal

**Affiliations:** UKM Medical Molecular Biology Institute, UKM Medical Centre, Universiti Kebangsaan Malaysia, Kuala Lumpur, Malaysia

**Keywords:** pharmacogenomics, precision medicine, colorectal cancer, DNA sequencing, gene variants, actionable target

## Abstract

Colorectal cancer (CRC) remains as one of the most common cause of worldwide cancer morbidity and mortality. Improvements in surgical modalities and adjuvant chemotherapy have increased the cure rates in early stage disease, but a significant portion of the patients will develop recurrence or advanced disease. The efficacy of chemotherapy of recurrence and advanced CRC has improved significantly over the last decade. Previously, the historical drug 5-fluorouracil was used as single chemotherapeutic agent. Now with the addition of other drugs such as capecitabine, irinotecan, oxaliplatin, bevacizumab, cetuximab, panitumumab, vemurafenib, and dabrafenib, the median survival of patients with advanced CRC has significantly improved from less than a year to the current standard of almost 2 years. However, the side effects of systemic therapy such as toxicity may cause fatal complications and have a major consequences on the patients’ quality of life. Hence, there is an urgent need for key biomarkers which will enable the selection of optimal drug singly or in combination for an individual patient. The application of personalized therapy based on DNA testing could aid the clinicians in providing the most effective chemotherapy agents and dose modifications for each patient. Yet, some of the current findings are controversial and the evidences are conflicting. This review aims at summarizing the current state of knowledge about germline pharmacogenomics DNA variants that are currently used to guide therapeutic decisions and variants that have the potential to be clinically useful in the future. In addition, current updates on germline variants conferring treatment sensitivity, drug resistance to existing chemotherapy agents and variants affecting prognosis and survival will also be emphasized. Different alteration in the same gene might confer resistance or enhanced sensitivity; and while most of other published reviews generally stated only the gene name and codon location, we will specifically discuss the exact variants to offer more accurate information in this mini review.

## Overview of Systemic Chemotherapy Regimens in Colorectal Cancer

The evolution of chemotherapy for colorectal cancer (CRC) was instigated with the development of 5-fluorouracil (5-FU) in 1957 (Heidelberger et al., 1957). Heidelberger et al. (1957) at the University of Wisconsin witnessed that tumor cells favored uracil for nucleic acid biosynthesis, and accurately hypothesized that a fluorouracil analog would inhibit tumor cell division by blocking the conversion of deoxyuridine monophosphate (dUMP) to deoxythymidine monophosphate (dTMP). The next landmark in CRC chemotherapy history was the discovery of 5-FU antitumor activity potentiation by leucovorin, which led to a large number of phase I and II clinical trials for metastatic CRC (mCRC) in the 1980s (Mini et al., 1990). Leucovorin is the folic acid in its reduced form and it is metabolized into tetrahydrofolate including 5,10-CH_2_-FH_4_ (Rustum, 1991). 5,10-CH_2_-FH_4_ is a cofactor that plays the role as a methyl donor for the dUMP conversion to dTMP by thymidylate synthase (TS) and is also important for interaction of FdUMP (one of the 5-FU active metabolites) with TS (Zaniboni, 1997). FdUMP, TS, and 5,10-CH_2_-FH_4_ form a covalent ternary complex whose stabilization leads to prolonged inhibition of TS activity and consequently, inhibition of DNA synthesis (Zaniboni, 1997). A meta-analysis of nine randomized clinical trials proved that leucovorin/5-FU combination increases the response rate from 11 to 23% compared to 5-FU alone in mCRC (Piedbois et al., 1992). Despite being a backbone to CRC treatment, 5-FU possess a short half-life and constantly require infusions. Prolonged infusion was cumbersome for patients and can result in medical complications (Johnston and Kaye, 2001). Therefore an oral formulation of the drug is deemed necessary. In 2005, capecitabine was sanctioned by the Food and Drug Administration (FDA) as an oral prodrug of 5-FU to be used as monotherapy in the adjuvant setting for treating advanced stage CRC (Hirsch and Zafar, 2011).

The standard practice for 5-FU dosing is based on patient’s body surface area (BSA); even though there is no significant correlation between the plasma clearance of 5-FU and BSA (Lee et al., 2016). An alternative pharmacokinetically guided (PKG) dosing strategy based on area under the concentration versus time curve (AUC) has remarkably improves the treatment response and lowered the risk of grade 3/4 toxicity for locally advanced CRC (Chen and Yee, 2016; Fang et al., 2016). However, further validation on this AUC-based PKG dosing focusing on prospective large-scale, randomized trials is still warranted (Chen and Yee, 2016). Tumor genetic makeup information, combined with AUC-based PKG dosing of 5-FU is foreseen to advance the development of personalized treatment in CRC patients further (Chen and Yee, 2016).

In the early 2000s, introduction of oxaliplatin and irinotecan as components of combination therapies for advanced disease marked another significant development in mCRC treatment (Gustavsson et al., 2015). Oxaliplatin, a platinum-containing agent, was discovered by Yoshinori Kidani in 1976 (Kidani et al., 1976, 1980) while irinotecan, a topoisomerase I inhibitor, was first discovered and synthesized by Yakult Honsha Ltd. in 1983 (Kunimoto et al., 1987). The effectiveness of irinotecan and oxaliplatin was first shown in patients that exhibited poor response to 5-FU/leucovorin, whereby 5-FU and leucovorin in combination with irinotecan (FOLFIRI) or oxaliplatin (FOLFOX) result in significantly increased response rates and improved survival compared to 5-FU/leucovorin alone (Douillard et al., 2000; MOSAIC study by André et al., 2004; NSABPC-07 study by Kuebler et al., 2007).

Another key development in the systemic management of mCRC was the advent of monoclonal antibodies targeting the vascular endothelial growth factor (VEGF) pathway or epidermal growth factor receptor (EGFR) (Cunningham et al., 2004; Hurwitz et al., 2004; Giantonio et al., 2007; Van Cutsem et al., 2007, 2009). Presently obtainable targeted agents active in mCRC include the anti-VEGF antibody (bevacizumab), the anti-EGFR antibodies (panitumumab and cetuximab), and the anti-VEGF agents (regorafenib and aflibercept). Due to these advancement in drug development, the median survival of CRC patients has significantly improved; yet, the side effects of systemic therapy such as toxicity, have become an important issue. Hence, there is an urgent need for biomarkers which could accurately predict each patient’s response to each of the drug to enable optimal dosing or to provide alternative treatment. **Table 1** summarizes the list of germline pharmacogenomics variants associated or potentially associated with adverse events following chemotherapy in CRC patients.

**Table 1:** List of pharmacogenomics germline variants associated or potentially associated with toxicity or drug response in CRC patients.

Gene	Variant	Treatment regimen	Number of subject	Reference
*DPYD*	IVS14+1G>A^†^ (c.1905+1G>A) rs3918290	Capecitabine, oxaliplatin and bevacizumab ± cetuximab	568	Deenen et al., 2011
		Fluoropyrimidines (5-FU, capecitabine, or tegafur–uracil)	7365	Meulendijks et al., 2015
		Capecitabine	2038	Deenen et al., 2016
	D949V^†^ (c.2846A>T) rs67376798	Capecitabine, oxaliplatin, and bevacizumab ± cetuximab	568	Deenen et al., 2011
		FOLFOX ± cetuximab	2886	Lee et al., 2014
		FOLFOX4 ± cetuximab	1545	Boige et al., 2016
	I560S^†^ (c.1679T>G) rs55886062	FOLFOX6	1	Dhelens et al., 2016
		Fluoropyrimidines (5-FU, capecitabine, or tegafur–uracil)	7365	Meulendijks et al., 2015
	V732I (c.2194G>A) rs1801160	FOLFOX4	1545	Boige et al., 2016
	C29R (c.85T>C) rs1801265	5-FU and folinic acid	1	Baskin et al., 2015
	M166V (c.496A>G) rs2297595	Capecitabine, oxaliplatin, and irinotecan + bevacizumab or cetuximab	64	Falvella et al., 2015
	I543V (c.1627A>G) rs1801159	5-FU and oxaliplatin	75	Zhang et al., 2007
*TYMS*	2R/2R genotype (rs45445694)	Radiation therapy (XRT), mitomycin C (MMC) and 5-FU	1	Wang et al., 2016
		Capecitabine and other fluorouracil-based regimens	927 in systematic literature review 4855 in meta-analysis	Rosmarin et al., 2014
		5-FU	90	Lecomte et al., 2004
	Q18Rfs^∗^42 (c.53_84del)	FOLFOX, FOLFIRI, FOLFIRI, and bevacizumab, panitumumab	1	Balboa-Beltrán et al., 2015
*ENOSF1*	c.742-227G>A rs2612091	Capecitabine	968 patients from the QUASAR2 clinical trial	Rosmarin et al., 2014
*MTHFR*	A222V (c. 667C>T) rs1801133	5-FU	544	Derwinger et al., 2009
	E429A (c.1298A>C) rs1801131	5-FU and radiotherapy	13	Thomas et al., 2011
*ABCB1*	I1145I (c.3435T>C) rs1045642	5-FU	67	Gonzalez-Haba et al., 2010
		Capecitabine	239	García-González et al., 2015
	G412G (c.1236C>T) rs1128503	Capecitabine	74	Gonzalez-Haba et al., 2010
			239	García-González et al., 2015
	A893S/T (c.2677G>T/A) rs2032582	Capecitabine	239	García-González et al., 2015
*CDA*	K27Q (c.79A>C) rs2072671	Capecitabine	239	García-González et al., 2015
	c.-92A>G rs602950	Capecitabine	430	Loganayagam et al., 2013
	c.-451C>T rs532545			

*^†^Clinically implemented. 5-FU, 5-fluorouracil; FOLFOX, fluorouracil, leucovorin, and oxaliplatin; FOLFIRI, folinic acid (leucovorin), fluorouracil and irinotecan; bFOL, bolus 5-FU, leucovorin and oxaliplatin.*

## DPYD

The most commonly discussed marker of 5-FU toxicity is dihydropyrimidine dehydrogenase (DPD), an enzyme encoded by the *DPYD*, a large gene (950 kb) spanning 4,399 nucleotides in 23 coding exons on chromosome 1p22 (Wei et al., 1998). *DPYD* catalyzes 5-FU inactivation into dihydrofluorouracil, typically in the liver (Caudle et al., 2013) and deactivates more than 85% of standard doses of 5-FU and capecitabine (Boige et al., 2016). Reduced or lack of DPD enzymatic activity affects approximately 5% of the overall population, subsequently extend the half-life of the drug, leading to excess accumulation of drug and toxicity (Lee et al., 2004). Besides, 3–5% of the population has an incomplete DPD deficiency due to sequence alterations in *DPYD* gene, which possibly limits the ability of the liver to fully metabolize fluorouracil, thereby resulting in toxicity (Boige et al., 2016; Dhelens et al., 2016).

Clinical Pharmacogenetic Implementation Consortium (CPIC) has proposed genotype-based dosage recommendation for 5-FU according to the presence of DYPD allelic variants IVS14+1G>A (^∗^2A allele), I560S (^∗^13 allele) and D949V (Offer et al., 2014). The CPIC proposes a substitute drug for homozygous patients and 50% starting dose reduction for heterozygous patients. For the ease of understanding and to be currently relevant, variants will be now referred to by the amino acid change for which they encode.

The splice site variant IVS14+1G>A is the most well-known single nucleotide polymorphism resulting in 5-FU toxicity. Located at the exon 14 intron border, this polymorphism leads to a splicing defect, causing skipping of the whole exon and produces a non-functional truncated protein (Van Kuilenburg et al., 1997). This splice variant is significantly associated with reduced 5-FU degradation rate (Gentile et al., 2016). It is strongly associated with a severe and life-threatening toxicity in treatment regimens which include capecitabine alone or in combination with others such as oxaliplatin, bevacizumab, cetuximab, 5-FU, or tegafur–uracil (Deenen et al., 2011, 2016; Meulendijks et al., 2015). The second well-known variant, I560S is remarkably infrequent in the general population, yet it has been constantly associated with reduced DPD activity (Offer et al., 2013a,b; Nie et al., 2017) and increased incidence of toxicity (Meulendijks et al., 2015; Dhelens et al., 2016). Clinical studies have also constantly proven the association between the third variant, D949V, and severe toxicity after chemotherapy that incorporated 5-FU. Patients with D949V necessitate significant capecitabine dose reduction due to severe toxicity (Deenen et al., 2011). Lee et al. (2014) also reported statistically significant associations between D949V and grade ≥3 adverse events in patients treated with FOLFOX alone or combined with cetuximab. More recently, Boige et al. (2016) confirmed the significant impact of D949V in patients treated with FOLFOX4 with or without cetuximab. Collectively, these three alleles are the only clinically implemented variants to date (Whirl-Carrillo et al., 2012).

Even though many studies has reported the significant association between these three biomarkers with reduced DPD activity and 5-FU toxicity, its prediction power is restricted by low minor allele frequency across the general population (Cross et al., 2010). Furthermore, ethnicity seems to be playing a role with certain variants significantly associated with toxicity or reduced enzyme activity in one population but not observed in other population, most likely due to differences in allelic frequency. These findings demonstrate a serious knowledge gap in pharmacogenetic biomarkers of 5-FU toxicity, which has been previously studied in populations of limited diversity.

In a substudy by PETACC-8 randomized phase III clinical trial, genotyping in 1,545 patients revealed the association of D949V and also V732I with adverse events following FOLFOX4 treatment with or without cetuximab (Boige et al., 2016). The significant association of V732I was further replicated in an independent cohort of 339 mCRC patients receiving FOLFOX4 in the FFCD 2000-05 phase III trial (Ducreux et al., 2011). The PETACC-8 is the only trial which demonstrated the association between V732I and fluorouracil-related adverse event so far.

There are many other *DPYD* variants reported but with weak evidence and conflicting data regarding the link between the variants with reduced 5-FU metabolism or increased 5-FU toxicity. For example, C29R (^∗^9A) was identified in a DPD-deficient patient (Vreken et al., 1997a) and shown to be catalytically inactive (Vreken et al., 1997b); however, clinical studies fail to demonstrate its relationship with toxicity (Caudle et al., 2013; Offer et al., 2014). Nevertheless, there is one case report linking C29R with ocular toxicity in a CRC patient when treated with 5-FU and folinic acid (Baskin et al., 2015). On the other hand, another study proposed that C29R may even has protective effect against 5-FU toxicity (Kleibl et al., 2009). Another variant, M166V, was revealed to be strongly linked with grade 3–4 toxicity in breast and gastroesophageal cancer patients, but not in CRC patients upon treated with a 5-FU-based therapy (Gross et al., 2008). Another three independent studies also unable to rectify a link between M166V and toxicity associated to 5-FU-based treatment (Amstutz et al., 2009; Deenen et al., 2011; Loganayagam et al., 2013). To date, the significant association between M166V and toxicity was only demonstrated by Falvella et al. (2015) who performed univariate and multivariate analysis involving 64 patients, in which the treatment regiments included capecitabine, oxaliplatin and irinotecan with bevacizumab or cetuximab. In another study involving 75 gastric and colon carcinoma patients, I543V (^∗^5) leads to the decreased enzyme activity and significant toxicity toward 5-FU (Zhang et al., 2007); however, the association was not significant in PETACC-8 randomized phase III clinical trial (Boige et al., 2016).

The ability of NGS technology in enabling sequencing of the whole coding sequence has resulted in discovery of various novel *DPYD* variants whose functions and association with toxicity remain unclear. Nevertheless, several studies have linked the novel genotype to the respective phenotype characteristics. For example, the three clinically implemented variants (IVS14+1G>A, I560S and D949V) were not detected in any of the 588 individuals of Somali or Kenyan ancestry living in central/southeast Minnesota (Elraiyah et al., 2017). However, via NGS, the authors identified several novel *DPYD* variants including I971Nfs which resulted in the complete lack of enzyme activity, and P86L, T793I, V941A, P1023S, A513V, and P237L, in which all lead to significantly reduced DPD activity.

## *Tyms* and *Enosf1*

Thymidylate synthase is considered to be a key intracellular target of fluoropyrimidines and is involved in dUMP conversion to dTMP, which is the exclusive source of thymidylate, a vital precursor for DNA synthesis (Heidelberger et al., 1957). Therefore, another candidate gene to predict 5-FU toxicity is the TS gene (*TYMS*), which is strongly inhibited by 5-FU (Shahrokni et al., 2009). This gene is located on the chromosome 18 at p11.32 which covers seven exons (Jakobsen et al., 2005). The *TYMS* promoter encompasses of a 28-bp sequence in the 5′-untranslated enhanced region (5′-UTR) and presented as a double-tandem repeat (2R) or a triple-tandem repeat (3R) (Huang et al., 2016).

Triple-tandem repeat (3R) and double-tandem repeat (2R) polymorphisms are associated with *TYMS* expression, 5-FU response and toxicity in a different manner. The 2R/2R genotype exhibits significantly lower TS mRNA levels compared to 3R/3R or 2R/3R (Pullarkat et al., 2001). Homozygous 2R/2R is significantly associated with increased susceptibility to 5-FU toxicity and drug sensitivity (Lecomte et al., 2004; Shahrokni et al., 2009). This fact is further strengthen by a meta-analysis in 4,855 patients by Rosmarin et al. (2014) which involved QUASAR2 and 16 other published studies. More recently, a case report on a CRC patient with homozygous 2R/2R receiving 5-FU therapy, mitomycin-C and adjuvant radiation who develop severe gastrointestinal toxicities and pancytopenia associated with 5-FU (Wang et al., 2016).

On the other hand, 3R polymorphism confers different effect compared to 2R. Pullarkat et al. (2001) suggest that 3R allele is responsible for 3.6 times higher *TYMS* in patients with mCRC compared to patients who were carrying 2R variant. Patients with the 3R/3R genotype had significantly less severe side effect and toxicity with 5-FU-based chemotherapy when compared to the 2R/2R genotype (Pullarkat et al., 2001). However, 3R/3R polymorphism will render the treatment ineffective, which subsequently lead to poor prognosis. Due to their distinct consequences, these two variations pose a dilemma in 5-FU-based cancer therapy; whereby high expression by 3R/3R leads to reduced drug sensitivity, whereas low expression by 2R/2R leads to the drug toxicity that may cause cessation of the therapy.

*TYMS* tandem repeat polymorphisms are the most studied alteration in this gene to date. However, novel mutation in coding regions have also begun to be revealed. For instance, Balboa-Beltrán et al. (2015) reported the first discovery of heterozygous germline stop codon mutation Q18Rfs^∗^42 in a mCRC patient which cause loss of function of one of the *TYMS* alleles, resulting in a truncated protein. Despite of a long survival (more than eight years after diagnosis), the patient developed severe toxicity under 5-FU-based therapy. Due to the absence of *DYPD* IVS14+1G>A, D949V, I560S and several other markers in relation to toxicity, the authors hypothesize that *TYMS* Q18Rfs^∗^42 could be the cause of severe toxicities suffered by the patient.

*ENOSF1* is a poorly characterized gene located adjacent to *TYMS*, and is hypothesized to code a protein and also an antisense transcript to *TYMS*, thus regulating its mRNA and/or protein expression (Dolnick et al., 2003). Rosmarin et al.’s (2014) identified a significant association between capecitabine toxicity and *ENOSF1* c.742-227G>A. The authors further concluded that the presence of this intronic variant was the cause of 5-FU toxicity reported in patients with functional *TYMS* but harboring 50 VNTR and 30 UTR polymorphisms (Rosmarin et al., 2014).

## MTHFR

Methylenetetrahydrofolate reductase is encoded by *MTHFR* gene and could also be an important predictor of response toward 5-FU. Methylenetetrahydrofolate reductase catalyzes the change of 5-10-methylenetetrahydrofolate, which is essential to the DNA synthesis, to 5-methyltetrahydrofolate by acting as a cofactor in conversion of dUMP to dTMP by TS (Jakobsen et al., 2005). In addition, this enzyme maintains the stability of the binding of 5-fluorodeoxyuridine monophosphate to TS, subsequently results in sustained inhibition of the enzyme.

Several single nucleotide polymorphisms (SNPs) are shown to influence the activity of *MTHFR*, and the variant with the significant association with 5-FU response in CRC is *MTHFR* A222V. It results in a substantial lower activity and thermolability of the enzyme in heterozygotes and homozygotes variant compared to the wild-type homozygotes (Sohn et al., 2004). Subsequently, a buildup of 5-10-methylenetetrahydrofolate is observed, and increased 5-FU sensitivity is anticipated. *In vitro* studies have identified the *MTHFR* A222V as a vital predictor of 5-FU response, where it changes intracellular folate distribution, accelerates cellular growth rate, increases TS activity and chemosensitivity of CRC cells to 5-FU (Sohn et al., 2004). A study by Jakobsen et al. (2005) proved the significant association of *MTHFR* A222V polymorphism with increased 5-FU response. The association was further demonstrated by Derwinger et al. (2009) who concluded that *MTHFR* A222V not only affect the 5-FU sensitivity, but also increased the risk of unwanted side-effects and subsequently affecting survival in stage III and stage IV CRC. Etienne-Grimaldi et al. (2010) showed that even though there was a significant association between *MTHFR* A222V and increase 5-FU response, this variant is not a toxicity predictor. On the other hand, Thomas et al. (2011) reported that *MTHFR* E429A and *MTHFR* diplotypes (for A222V and E429A) were significantly connected to toxicity when only 5-FU was used. In contrast Etienne-Grimaldi et al. (2010) showed that there was no association between these biomarkers and drug response.

Another study using capecitabine monotherapy and capecitabine-irinotecan in mCRC reported conflicting results (van Huis-Tanja et al., 2013). The authors showed that *MTHFR* E429A and A222V genotypes were not associated with efficacy or toxicity in mCRC patients. Comparisons among different genotypes of *MTHFR* 1298 locus revealed that CC homozygotes exhibited greater incidence of grade 3–4 diarrhea compared with AC or AA individuals but only at borderline significance. Therefore, it remains controversial whether *MTHFR* polymorphisms can possibly predict toxicity or 5-FU response in patients treated with fluoropyrimidines.

## ABCB1

The *ABCB1* gene, located on chromosome 7 (Schrickx and Fink-Gremmels, 2008) belongs to drug transporter gene family. T carriers of *ABCB1* I1145I treated with 5-FU had a significantly higher risk of diarrhea (García-González et al., 2015), while the same genotype conferred a lower risk of hand-and-foot syndrome when treated with capecitabine (Gonzalez-Haba et al., 2010), despite these two drugs belong in the same class (fluoropyrimidines). The association between *ABCB1*^∗^1 haplotype [1236C, 2677G (893Ala), and 3435C] with severe overall toxicity was significant in 239 capecitabine-treated CRC patients (García-González et al., 2015).

## CDA

The *CDA* gene, located on chromosome 1 (Saccone et al., 1994), encodes an enzyme in the pyrimidine salvage pathway and is important for metabolism of antitumor cytosine nucleoside analogs, leading to 5-FU activation (Lou et al., 2016). Despite scarce evidence, the relationship between *CDA* promoter variants and 5-FU toxicity in CRC has been demonstrated. *CDA* c.-92A>G and c.-451C>T variants were significantly associated with grade 2–4 diarrhea (Loganayagam et al., 2013), while exonic K27Q AA is significantly associated with ≥3 overall toxicity (García-González et al., 2015).

## Bed-To-Bedside Challenges, Future Recommendation, and Conclusions

In summary, we have reviewed the current information of potential pharmacogenetics gene variants that could help the oncologists in making therapeutic decisions. The clinical utility of *DPYD* IVS14+1G>A, D949V, and I560S have been demonstrated in a number of large prospective/retrospective studies and clinical trials; however, the discrepancies of other *DPYD* variants among multistudies could be due to small cohorts that include diverse stages and treatment regimens. Lesser evidence is available for *TYMS, ENOSF1, MTHFR, ABCB1*, and *CDA*; nevertheless, at least one study which includes hundreds of CRC patients has reported the significant association among the altered genes with adverse events and this information should not be disregarded. Nonetheless, the role of variants in *TYMS, ENOSF1, MTHFR, ABCB1*, and *CDA* genes still warrant for additional validation in larger cohort.

The clinical application of precision medicine is hindered by several disagreements such as the unwillingness of clinicians, ministry of health, and insurance organizations to adopt this into clinical practice. Unless we overcome these major hurdles, precision medicine will have a long way to go. While the former president of the United States, Barack Obama, had declared the Precision Medicine initiative in 2015, other countries have yet to embark on a similar initiative especially so in the developing nations, hence more effort still need to be done to increase awareness of precision medicine.

The role of ethnic differences in drug response or toxicity is accepted as a crucial factor responsible for inter-individual variation in anticancer drug sensitivity (O’Donnell and Dolan, 2009; Mohelnikova-Duchonova et al., 2014). A toxicity biomarkers in Caucasian might differ from Asian or African descendant patients. Therefore, identification of ethnic-specific biomarkers for drug response is imperative. Information compiled in this review could be applied in designing a targeted gene panel for personalized medicine in CRC. Genotyping of these genes in advance before chemotherapy could help to lessen the adverse reactions in CRC patients.

## Author Contributions

N-SA, NFM, and S-NA drafted this manuscript. N-SA and RJ were responsible for idea conception, critical evaluation, and manuscript review.

## Conflict of Interest Statement

The authors declare that the research was conducted in the absence of any commercial or financial relationships that could be construed as a potential conflict of interest.
